# Spatiotemporal Dynamics of Habitat Ecological Intactness and Responses of Wild Giant Panda Populations in Sichuan, China

**DOI:** 10.3390/biology15201799

**Published:** 2026-10-09

**Authors:** Qinlong Dai, Junmu Yue, Yue Wang, Xiaodong Gu, Wei Wei

**Affiliations:** 1 Liziping Giant Panda Ecology and Conservation Observation and Research Station of Sichuan Province, Nanchong 637009, China; 2 Shimian Management and Protection Station, Giant Panda National Park, Ya’an 625400, China; 3 College of Giant Panda, China West Normal University, Nanchong 637009, China; 4 Sichuan Forestry and Grassland Bureau, Chengdu 610081, China

**Keywords:** anthropogenic disturbance, land-use change, human footprint, biodiversity conservation, ecological restoration, Liangshan mountains

## Abstract

**Simple Summary:**

Giant pandas are an iconic flagship species for global biodiversity conservation, and protecting their habitat is key to their survival. However, human activities such as road construction, farming, and mining continue to change these habitats. In this study, we examined how the ecological intactness of the giant panda habitat (the degree to which it remains free from human disturbance) has changed over the past 40 years in the Sichuan section of the Giant Panda National Park and the nearby Liangshan Mountains. We found that the habitat is still in relatively good condition overall but has been slowly degrading; among the components of the intactness index, land-use type and road construction contributed most to the spatial differences in habitat intactness. We also found that wild giant pandas are concentrated in areas with less human disturbance, although habitat intactness alone does not predict how many pandas an area can support. These findings can help managers target conservation efforts more effectively and provide a scientific basis for deciding whether the Liangshan Mountains should be incorporated into the national park, giving more wild giant pandas a safer home.

**Abstract:**

Habitat degradation caused by anthropogenic disturbance is a major threat to global biodiversity, yet the spatiotemporal dynamics of the ecological intactness of giant panda habitat and the associated population responses remain poorly quantified. We focused on the Sichuan section of the Giant Panda National Park and the Liangshan Mountains. Using multi-source anthropogenic disturbance data and three national giant panda surveys, we integrated fuzzy overlay analysis, Theil–Sen trend analysis, Geodetector, and the Mann–Whitney U test to analyze the spatiotemporal dynamics, contributing disturbance components, and population responses of habitat intactness. The mean habitat intactness in 2020 was 6.70. Intactness declined slowly during 1980–2020, and 73.7% of the habitat remained stable. Among the disturbance components of the intactness index, land-use type and road construction contributed most to its spatial variation, and the joint contribution of any two interacting components exceeded that of either component alone. The intactness of giant panda occurrence sites was higher than that of systematic background points (significant in five of the six survey-by-region comparisons) and was higher in the Liangshan Mountains than in the national park. However, intactness was not significantly correlated with population density. These results indicate that wild giant pandas are concentrated in high-intactness habitat, although intactness alone does not predict population density, and they provide empirical evidence for intactness-oriented conservation management.

## 1. Introduction

Human activities such as agricultural expansion, infrastructure construction, resource extraction, and urbanization have modified more than 70% of the global land surface, causing habitat loss, landscape fragmentation, and biodiversity decline [1,2]. Earth’s ecosystems are now at risk of a sixth mass extinction [3]: land use has already reduced local terrestrial biodiversity beyond the level proposed as a safe planetary boundary [4], and remaining wilderness areas are being eroded rapidly [5]. Reducing the intensity of anthropogenic modification and maintaining ecological intactness have become priority goals of the global biodiversity conservation framework [6,7] and important tasks in the development of China’s national park system [8]. Ecological intactness refers to the degree to which an ecosystem retains its natural baseline and remains free from anthropogenic modification [9,10]. Ecosystems with high intactness provide irreplaceable ecosystem services and serve as important refuges for endangered wildlife [11].

The giant panda (*Ailuropoda melanoleuca* (David, 1869)) is an umbrella species endemic to the mountains of southwestern China. Following decades of conservation investment represented by the establishment of nature reserves, the Natural Forest Protection Program, and the Grain for Green Program [12,13], the wild giant panda population has recovered, and the IUCN Red List has downlisted the species from Endangered to Vulnerable [14,15,16]. Wild giant pandas are currently divided into 33 local populations, 18 of which comprise fewer than 10 individuals [17,18,19] and face a high risk of extinction owing to habitat degradation and fragmentation.

Quantitative assessments of ecological intactness are mainly based on the spatial gradients of anthropogenic disturbance. The Human Footprint Index quantifies the integrated intensity of human modification at the global scale [1,2,20]; the Biodiversity Intactness Index measures the degree to which ecosystems retain their natural state from the perspective of species abundance [9,10]; and Theobald [21] and McGarigal et al. [22] further developed landscape-scale ecological integrity indices that integrate the intensity and influence range of multiple anthropogenic disturbances into spatially continuous assessment layers. Globally, only about 40% of remaining forests still have high ecosystem integrity [23]. These methods have been applied to assess disturbance from land-use change [24] and to identify conservation priority areas. In the giant panda region, Luo et al. [25] assessed the spatiotemporal change in the ecological intactness of the Giant Panda National Park and its adjacent areas but did not address the responses of wild giant panda populations to intactness gradients or include a comparison with mountain ranges outside the national park.

To integrate the various overlapping and separately managed protected areas within the giant panda’s distribution range, China launched the pilot program for the Giant Panda National Park system in 2017 and officially established the park in 2021 as one of China’s first national parks. The park spans Sichuan, Shaanxi, and Gansu provinces and covers a total area of approximately 27,134 km^2^. It integrates the existing giant panda nature reserves, forest parks, and scenic areas and encompasses the main giant panda habitat of the Minshan, Qionglai, Daxiangling, Xiaoxiangling, and Qinling Mountains [26,27]. The park aims to mitigate habitat fragmentation, connect isolated populations, and maintain the ecological intactness of giant panda habitat across these mountain ranges [26,27,28,29]. The Liangshan Mountains lie outside the park and are a potential expansion area of the national park system; their intactness dynamics and population responses can provide a basis for optimizing the park’s boundary.

At the southern edge of the giant panda’s distribution range, the Liangshan Mountains face pronounced human–environment conflicts: local communities depend heavily on forest resources, grazing pressure is high, the human population is growing rapidly, and household incomes are low. Giant panda populations in this region are small, their habitat is highly fragmented, and the intensity of cumulative anthropogenic disturbance is high [30]. Existing studies of giant panda habitat have focused mainly on habitat suitability assessment, landscape fragmentation, and vegetation characteristics [31,32]. Although the conservation of ecological intactness has been incorporated into the planning documents of the Giant Panda National Park, the spatiotemporal dynamics of habitat ecological intactness over long time scales and the responses of wild giant panda populations to changes in intactness remain poorly quantified [25].

This study addresses three scientific questions: (1) What were the spatiotemporal patterns of habitat ecological intactness in the Sichuan section of the Giant Panda National Park and the Liangshan Mountains during 1980–2020? (2) Which anthropogenic disturbance components contribute most to the spatial variation in habitat intactness, and how do they interact? (3) How do giant panda occurrence sites and population density respond to spatial differences in habitat intactness? Answering these questions can provide scientific support for intactness-oriented giant panda conservation and inform decisions on whether the Liangshan Mountains should be incorporated into the Giant Panda National Park system in the future.

## 2. Materials and Methods

### 2.1. Study Area

The study area comprises the Sichuan section of the Giant Panda National Park and the Liangshan Mountains outside the park. The Giant Panda National Park harbors approximately 72% of China’s wild giant pandas [26,27,28,29]. Its Sichuan section covers 20,177 km^2^ in the transition zone from the Sichuan Basin to the Qinghai–Tibet Plateau and spans the Minshan, Qionglai, Daxiangling, and Xiaoxiangling Mountains. Elevation ranges from 582 to 6132 m, with complex terrain, pronounced climatic differentiation, and rich wildlife resources.

The Sichuan section has a subtropical monsoon climate that varies strongly with topography: the east is influenced by the southeast monsoon, the southwest by the southwest monsoon (wet summers and dry winters), and the northwestern highlands have a plateau climate with long, cold winters. Annual precipitation exceeds 800 mm in the east and reaches up to 1700 mm along the western margins, whereas mean annual temperature is above 16 °C in the basins but below 16 °C in the mountains [26,27]. The vertical zonation of vegetation is well-developed, ranging from subtropical evergreen broadleaved forests below 1600 m, through mixed broadleaf–conifer forests at 1600–2600 m, to subalpine conifer forests at 2600–3600 m and alpine meadows and shrublands above 3600 m. The region lies within the Hengduan Mountains biodiversity hotspot and harbors more than 5600 plant species and over 1000 terrestrial vertebrate species, including endangered mammals such as the golden snub-nosed monkey (*Rhinopithecus roxellana* (Milne-Edwards, 1870)), the snow leopard (*Panthera uncia* (Schreber, 1775)), and the Sichuan takin (*Budorcas tibetanus* Milne-Edwards, 1874) [26,27,30].

**Figure 1:**
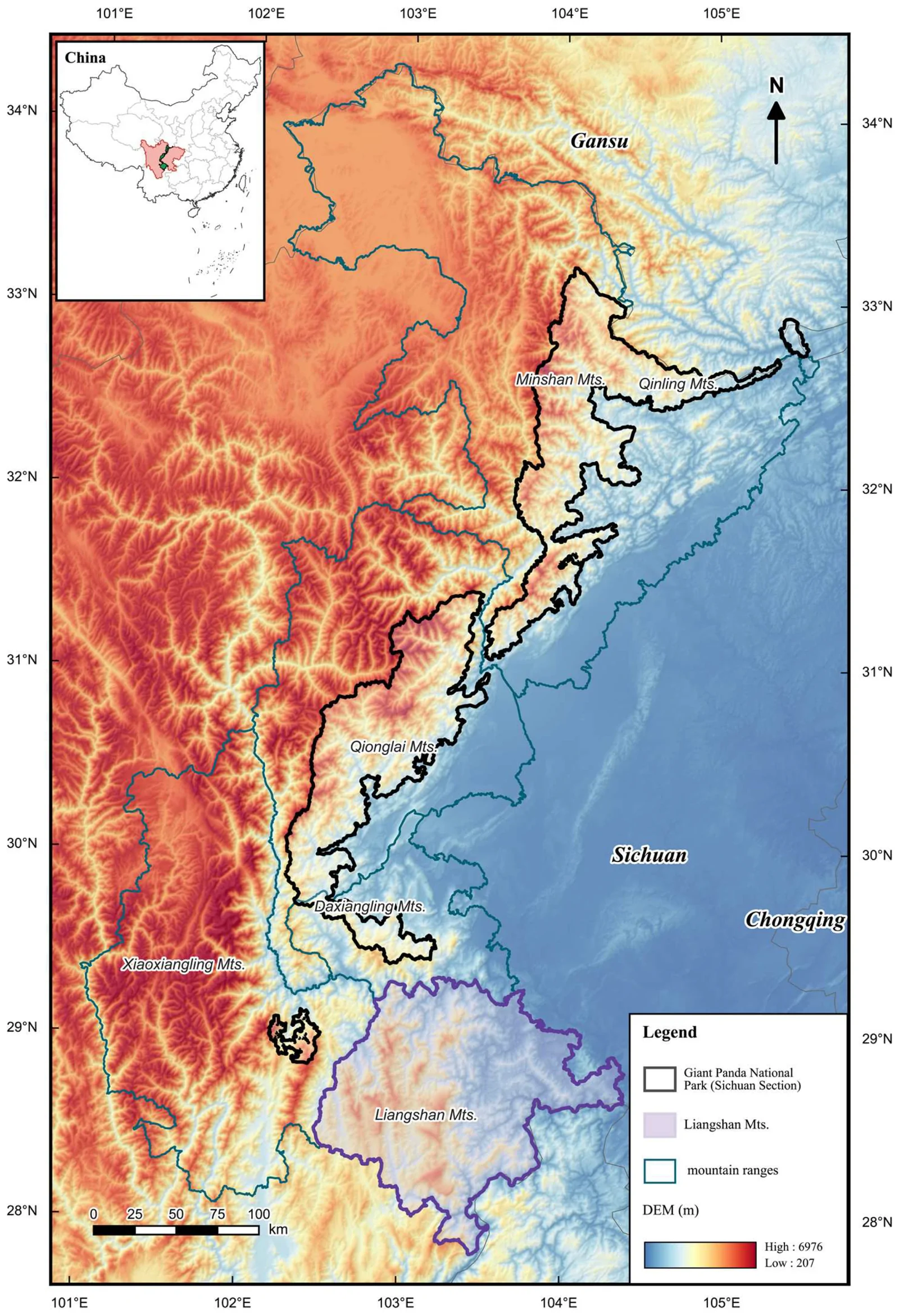
Geographic location of the study area in Sichuan Province, China. The six mountain ranges inhabited by the giant panda (*Ailuropoda melanoleuca* (David, 1869))—the Minshan, Qionglai, Daxiangling, Xiaoxiangling, Liangshan, and Qinling Mts. (teal boundaries)—constitute the study framework. Within them, the Giant Panda National Park (Sichuan Section), including the Liziping National Nature Reserve (NNR), is highlighted by the bold black boundary; the Liangshan Mts. (purple), a candidate range evaluated for potential inclusion in the park, are highlighted separately. Background shading shows terrain derived from the SRTM digital elevation model (DEM, ~90 m resolution), with colors ranging from blue (low elevation) to red (high elevation). The inset shows the location of Sichuan Province (red) and the study area (green) in China.

The Liangshan Mountains cover approximately 15,722 km^2^ in southwestern Sichuan. Local livelihoods depend heavily on agriculture and animal husbandry, and the habitat is subject to persistent anthropogenic disturbance. This region supports approximately 7% of China’s wild giant pandas, which are divided into multiple small populations. An overview of the study area is shown in Figure 1.

The Liangshan Mountains have a mild subtropical monsoon climate with distinct wet and dry seasons and pronounced vertical climatic zonation. Annual precipitation ranges from about 1000 mm in the drier western Daliangshan to 1600–2000 mm in the eastern Xiaoliangshan within the so-called Huaxi rain-screen belt [30]. Vegetation zonation includes broadleaved forests below 2600 m, cool-temperate conifer forests at 2600–3800 m, and meadows and alpine shrublands above 3800 m [30]. In addition to the giant panda, the region supports other threatened species such as the red panda (*Ailurus fulgens* F. Cuvier, 1825), the forest musk deer (*Moschus berezovskii* Flerov, 1929), and the clouded leopard (*Neofelis nebulosa* (Griffith, 1821)) [30].

### 2.2. Data Sources

Three types of datasets were used in this study. The first comprised giant panda distribution data from the Second (1985–1988), Third (1999–2003), and Fourth (2011–2014) National Giant Panda Surveys [17,18]. The first national survey was excluded from the analysis because of insufficient methodological precision and the lack of accurate geographic locations. The three surveys yielded 791, 3098, and 1846 giant panda occurrence sites, respectively, which were divided into two groups according to their location: the Sichuan section of the Giant Panda National Park and the Liangshan Mountains. Second-survey sites were generated from 5 km × 5 km distribution grids and the literature records, following the approach of previous national-survey analyses [17,28], whereas third- and fourth-survey sites were obtained directly from the respective survey databases. By region, 641 s-survey sites were located in the Sichuan section of the Giant Panda National Park and 150 in the Liangshan Mountains; the corresponding numbers were 2712 and 386 for the third survey and 1620 and 226 for the fourth survey.

The second dataset comprised land use and cover data from the China National Land Use/Cover Change dataset (CNLUCC) at 30 m resolution [33,34], for the years 1980, 1990, 2000, 2010, and 2020, covering six first-level classes and 25 s-level classes; its high interpretation accuracy makes it suitable for capturing long-term regional land-use change.

The third dataset comprised vector data on anthropogenic disturbances, including roads, hydropower and water conservancy projects, and mining sites, obtained from local archives, multi-year field survey records of nature reserves, and the disturbance registers of the national giant panda survey reports. The intensity levels and distance-decay influence ranges of different disturbance sources were determined with reference to published studies and verified in the field. A digital elevation model (DEM) with a resolution of approximately 90 m was used for the elevation-gradient analysis, and the boundaries of local populations and protected areas were derived from the Fourth National Giant Panda Survey [18,19]. The main input datasets are summarized in Table 1.

### 2.3. Ecological Intactness Calculation

In this study, ecological intactness refers to the degree to which habitat retains its natural state under remotely detectable anthropogenic modification pressures. For different types of disturbance sources (roads of different classes, hydropower and water conservancy projects of different scales, mines of different scales, and various land-use types), anthropogenic disturbance intensity was assigned values ranging from 0 to 10 according to the disturbance type and the Euclidean distance to the disturbance source, with higher values indicating stronger anthropogenic modification [21,22,35]. The intensity assignments followed the impact evaluation indicators developed by Theobald [21], and the maximum influence ranges of each disturbance type were determined through field surveys in the study area and adjusted with reference to the assignment standards of Luo et al. [25] and Li et al. [30] for similar disturbances. These assignments are consistent with the distance-decay disturbance regimes typical of the steep mountain valleys of southwestern China, where settlements, roads, and other infrastructure concentrate in the valleys and their effects attenuate rapidly with distance and elevation [21,22,25,30]. Because no independent measurements of disturbance intensity are available to calibrate these assignments, the absolute intactness scores should be regarded as estimates whose values depend on the assumed parameters; the spatial pattern of intactness and the contrast between the two sub-regions are, however, internally consistent and comparable under the same parameterization, because the same assignment scheme was applied uniformly across all years and areas. Roads were classified into expressways, railways, and first- to fourth-class roads, with a maximum influence range of 3 km; intensity values were assigned across four distance bands (0–100, 100–500, 500–1000, and 1000–3000 m; Table S1). Hydropower and water conservancy projects were classified into three levels (small/medium, large, and extra-large) according to installed capacity or project scale. The area within 500 m of the project center was treated as the dam zone, and intensity values were assigned outward across four distance bands (500–1000, 1000–1500, 1500–2500, and 2500–5000 m; Table S2). Mines were classified as small or large using a 1 km^2^ occupied-area threshold, and intensity values were assigned outward from the mine boundary across four distance bands (0–200, 200–500, 500–800, and 800–1000 m; Table S3). Intensity values for land use were assigned to each of the 25 s-level CNLUCC classes (Table S4). Disturbances such as grazing, bamboo shoot collection, medicinal herb collection, fire, and hunting were not included in the assignment system because quantitative spatial data were unavailable; this omission is particularly relevant for the Liangshan Mountains and high-elevation areas, where grazing and non-timber forest product collection are widespread, and it is discussed as a limitation in Section 5.

The integrated value of anthropogenic modification under multiple stressors, HM, was calculated using the fuzzy overlay function:

(1) $$HM=1-\prod_{i\;=\;1}^{n}\left(1-F_{i}\right)$$

where HM is the integrated score of all types of anthropogenic disturbance in the study area; F_i_ is the normalized score (ranging from 0 to 1) of the *i*-th type of anthropogenic disturbance; and *n* is the number of disturbance types. The habitat intactness score IS was calculated as follows:

(2) $$IS=10\times \left(1-HM\right)$$

Combining Equations (1) and (2), the final habitat intactness score was calculated as:

(3) $$IS=10\times \prod_{i\;=\;1}^{n}\left(1-F_{i}\right)$$

IS ranges from 0 to 10, where 10 represents habitat free of detectable anthropogenic disturbance and 0 represents a completely human-modified landscape. The calculations were performed with the Raster Calculator module in ArcGIS 10.8 (ESRI, Redlands, CA, USA).

### 2.4. Trend Analysis of Habitat Intactness

The Theil–Sen median slope estimator combined with the Mann–Kendall nonparametric significance test [36,37,38] was used to analyze the long-term trend of habitat intactness during 1980–2020 on a pixel-by-pixel basis. Compared with ordinary least squares regression, the Theil–Sen median method is more robust to outliers and was calculated as follows:

(4) $$\beta ={Median}_{i\;<\;j}\left(\frac{{IS}_{j}-{IS}_{i}}{j\;-\;i}\right)$$

where IS_i_ and IS_j_ are the habitat intactness scores in years *i* and *j*, respectively, and *β* is the median of the slopes of all *n*(*n* − 1)/2 year pairs; *β* > 0 indicates an increasing trend of intactness, and *β* < 0 indicates a decreasing trend. The Mann–Kendall test statistic was calculated as follows:

(5) $$S=\sum_{i\;=\;1}^{n\;-\;1}\sum_{j\;=\;i\;+\;1}^{n}sgn\left({IS}_{j}-{IS}_{i}\right)$$

(6) $$sgn\left(x\right)=\left\{\begin{array}{c} 1\;\;x>0\\ 0\;\;x=0\\ -1\;\;x<0 \end{array}\right.$$

(7) $$Var\left(S\right)=\frac{n\left(n-1\right)\left(2n+5\right)}{18}$$

(8) $$Z=\left\{\begin{array}{c} \frac{S-1}{\sqrt{Var\left(S\right)}}\;\;S>0\\ 0\;\;S=0\\ \frac{S+1}{\sqrt{Var\left(S\right)}}\;\;S<0 \end{array}\right.$$

where n is the length of the time series, and a two-tailed test was applied. Based on the median slope β and the Z statistic of the Mann–Kendall test, each pixel was classified into one of seven trend categories: when β > 0, Z > 1.96 indicates a significant increase, 1.65 < Z ≤ 1.96 a marginally significant increase, and Z ≤ 1.65 a non-significant increase; β = 0 indicates basic stability; and when β < 0, the same thresholds on |Z| define non-significant, marginally significant, and significant decreases. Although the series contains only five observations, the Theil–Sen median slope is a robust nonparametric estimator that remains reliable for short series and is widely used in five-period land-use change studies. The Mann–Kendall test is distribution-free and, with the two-tailed Z thresholds stated above, provides a consistent significance benchmark. Because significance tests on five points have limited statistical meaning at the pixel level, the magnitude and direction of β were treated as the primary evidence of change and the significance classes as secondary; accordingly, the trend categories are used to map the spatial pattern of change, whereas the magnitude of change is emphasized in the interpretation of the results.

### 2.5. Component Contribution Analysis Using Geodetector

Because the intactness index is constructed from the same disturbance layers, the Geodetector analysis in this study does not aim to identify independent external drivers of intactness; rather, it quantifies the relative contribution of each disturbance component layer to the spatial heterogeneity of the resulting intactness index [39,40]. To this end, five component layers of the intactness index were used as stratification variables: land-use type, road construction, hydropower construction, water conservancy construction, and mining. Land-use type was stratified by its second-level classes; the other four layers were discretized into the integer disturbance-intensity levels (0–10) of their respective assignment layers. All five stratification variables were input into the factor and interaction detectors, and the explanatory power *q* of each layer for the spatial heterogeneity of ecological intactness was quantified as follows:

(9) $$q=1-\frac{SSW}{SST}$$

(10) $$SSW=\sum_{h=1}^{L}N_{h}\sigma_{h}^{2},\;\;\;SST=N\sigma^{2}$$

where *h* = 1, 2, …, *L* denotes the factor strata; N_h_ and *N* are the numbers of pixels in stratum *h* and in the entire study area, respectively; *σ*_h_^2^ and *σ*^2^ are the variances of the intactness score in stratum *h* and in the entire study area, respectively; and SSW and SST are the within-stratum sum of squares and the total sum of squares for the entire study area, respectively. *q* ranges from 0 to 1, with larger values indicating stronger explanatory power of the factor. The interaction detector module was used to identify pairwise interaction effects between factors and to determine whether the explanatory power of two interacting factors was enhanced or weakened relative to their individual effects.

### 2.6. Statistical Analysis of Giant Panda Population Responses

Each giant panda occurrence site was matched to the intactness layer of the year nearest to the corresponding survey period (second survey: 1990 layer; third survey: 2000 layer; fourth survey: 2010 layer), and the habitat intactness value at each site was then extracted. Because the second-survey locations were reconstructed from 5 km × 5 km distribution grids and literature records rather than precise point records, their intactness values carry greater positional uncertainty than those of the third- and fourth-survey sites, and cross-survey comparisons were interpreted in light of this difference in spatial precision. As a regional reference, systematic background points were generated by overlaying a 5 km × 5 km fishnet on each sub-region and taking the cell centers (818 points in the national park and 634 points in the Liangshan Mountains); the same background points were used for all three survey periods. Mann–Whitney U tests [41] were performed in SPSS 27.0 (IBM Corp., Armonk, NY, USA) to compare (1) the intactness of giant panda occurrence sites between the national park and the Liangshan Mountains by survey period and (2) the intactness of occurrence sites versus that of systematic background points by study sub-region. Differences in intactness between the two zones within each elevation band (<1000, 1000–2000, 2000–3000, and >3000 m) were summarized as the mean ± SD of the 30 m pixels and were compared descriptively in terms of mean differences and variability; formal significance tests were not applied to these pixel-level comparisons, because neighboring pixels are spatially autocorrelated and such tests would yield optimistic *p*-values.

Local population units and nature reserves with more than 90% of their extent within the study area were selected. Using data from the Fourth National Giant Panda Survey, Pearson correlation analyses were conducted between mean habitat intactness and giant panda population density at the local-population and protected-area scales. The Theil–Sen and Mann–Kendall trend analyses were implemented in MATLAB R2019b (MathWorks, Natick, MA, USA); the Geodetector analyses were run in R version 4.4.1 (R Foundation for Statistical Computing, Vienna, Austria) using the GD package [39,40]; the spatial calculation and mapping of intactness were performed in ArcGIS 10.8 (ESRI, Redlands, CA, USA), which was also used to produce the study-area map; and the statistical graphics were produced in Python 3.14.2 with the matplotlib library.

## 3. Results

### 3.1. Current Status of Habitat Ecological Intactness in 2020

The study area covers a total of 36,472 km^2^, comprising the Sichuan section of the Giant Panda National Park (including the Liziping National Nature Reserve) and the Liangshan Mountains. The mean habitat ecological intactness across the entire region was 6.70; 72.0% of the region had intactness scores ≥ 7.0, whereas 22.4% had scores ≤ 6.0 and thus experienced relatively strong anthropogenic modification. Intactness increased with elevation, consistent with the lower intensity of anthropogenic disturbance at higher elevations.

**Figure 2:**
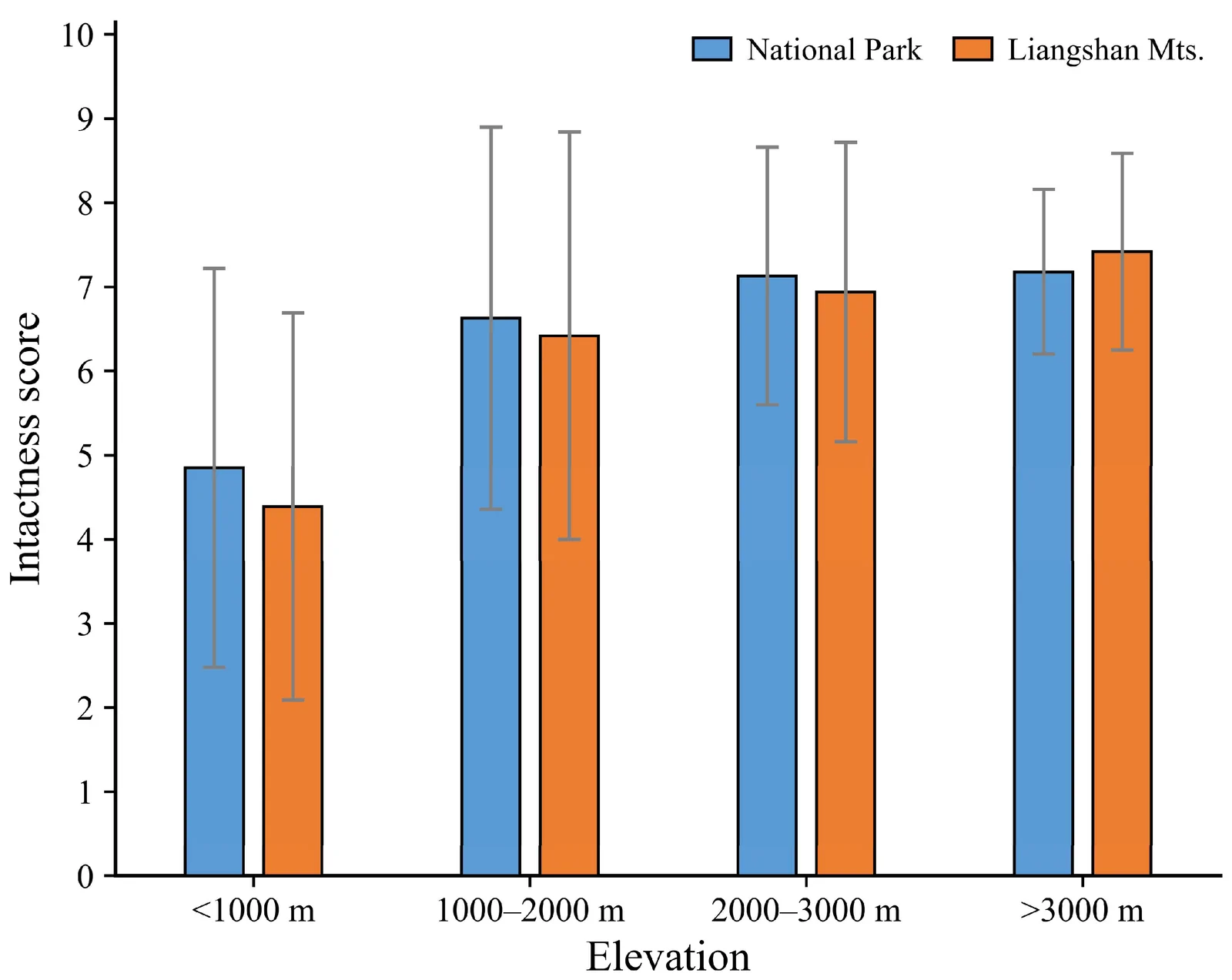
Ecological intactness scores (mean ± SD) of giant panda habitat along elevation gradients (<1000 m, 1000–2000 m, 2000–3000 m, >3000 m) in the Giant Panda National Park and the Liangshan Mountains. The statistical unit is the 30 m pixel; because neighboring pixels are spatially autocorrelated, the two regions are compared in terms of mean differences and variability rather than statistical significance.

The mean intactness of the Sichuan section of the Giant Panda National Park was 7.04 ± 1.61, higher than that of the Liangshan Mountains (6.26 ± 2.34; Table 2). Below 3000 m in elevation, habitat intactness in the national park was higher than that in the Liangshan Mountains, whereas above 3000 m the intactness score of the Liangshan Mountains was slightly higher (Figure 2). This contrast may be biased because the dataset did not capture diffuse anthropogenic disturbances (Section 2.3).

### 3.2. Spatiotemporal Dynamics of Habitat Intactness During 1980–2020

**Figure 3:**
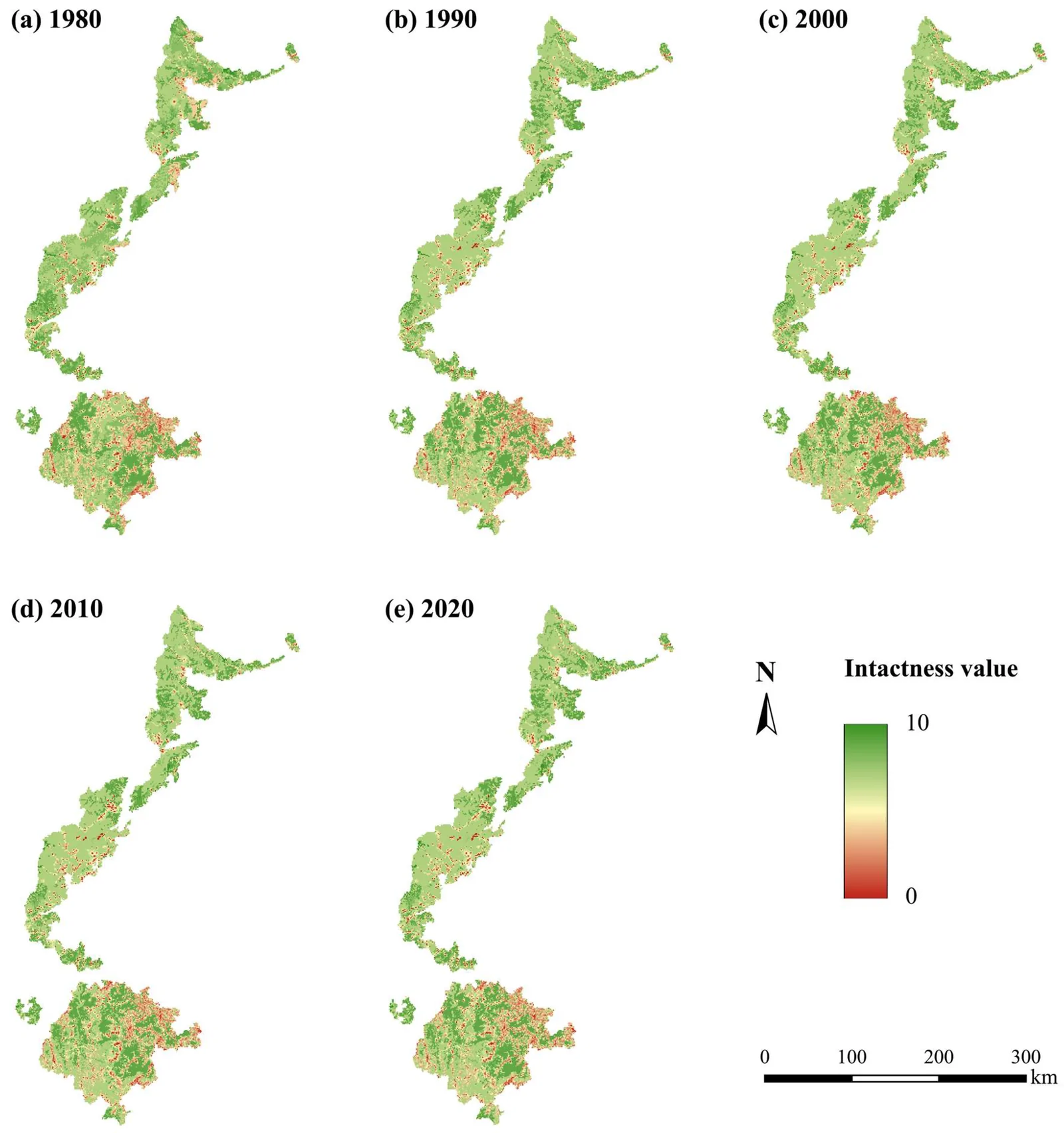
Spatiotemporal distribution of habitat ecological intactness (IS) from 1980 to 2020. The color scale indicates the intactness value (0–10), with higher values representing better-preserved habitat.

**Figure 4:**
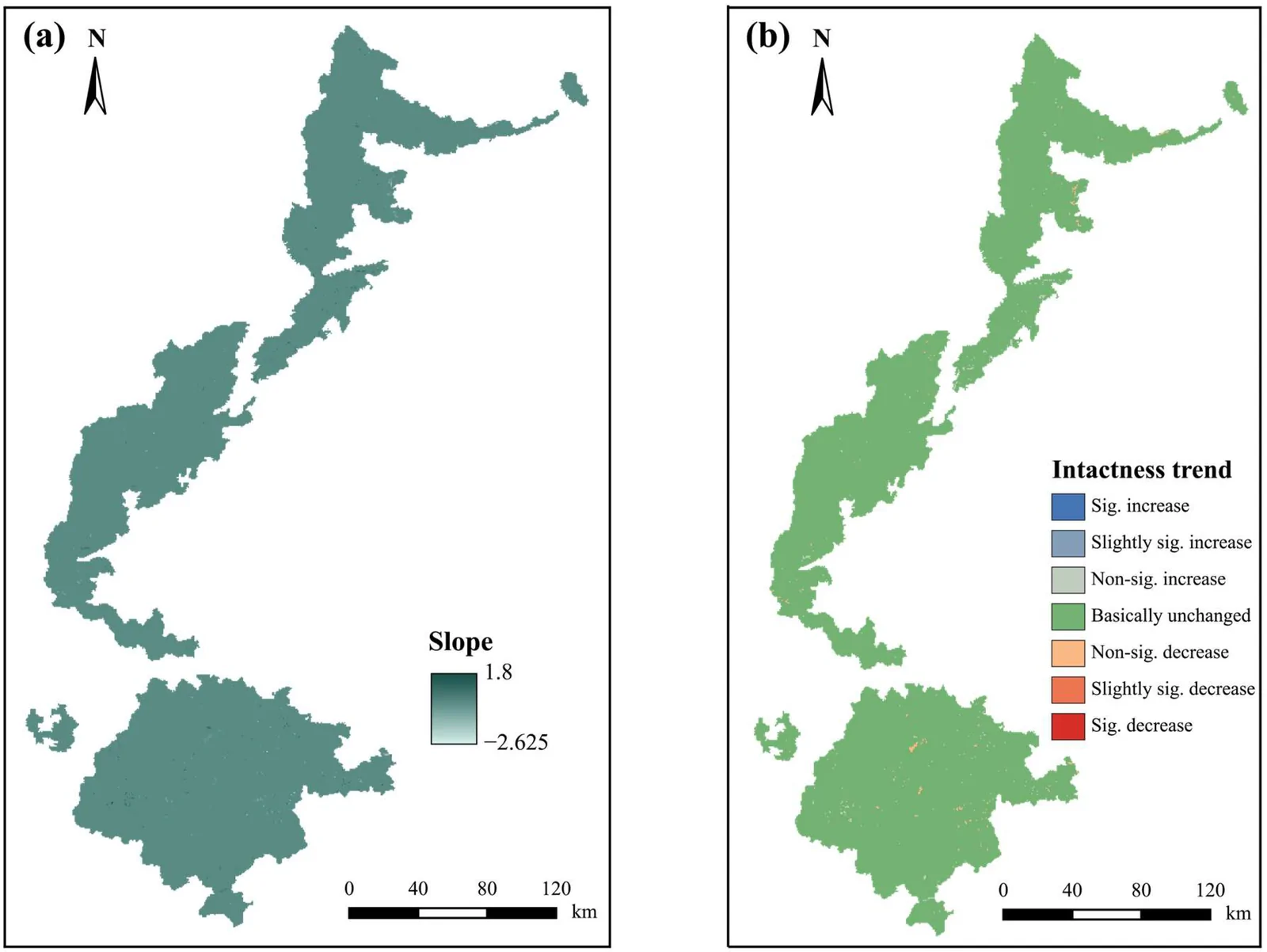
Spatial distribution of pixel-scale trends in habitat ecological intactness over the study period: (**a**) Theil–Sen median slope (*β*) of per-pixel IS (range: −2.63 to 1.83); (**b**) trend categories based on significance (Sig., significant; slightly sig., marginally significant; Non-sig., not significant).

During 1980–2020, the habitat intactness of the study area declined slowly overall, with the regional mean decreasing from 6.73 ± 2.06 in 1980 to a minimum of 6.69 ± 2.00 in 1990–2000 and then rebounding slightly to 6.70 ± 2.00 in 2010, where it remained in 2020 (Figure 3); the total range of the mean over the four decades was only 0.04 on the 0–10 scale, indicating that stability with a slight net decline was the dominant signal. The trend classification results (Figure 4) showed that 73.7% of the habitat remained stable in intactness; 0.4% showed a decreasing trend (marginally significant and non-significant decreases combined); 0.5% showed an increasing trend (marginally significant and non-significant increases combined); and significant increases and significant decreases each accounted for less than 0.01% of all pixels. Because the significance classes are derived from only five observations per pixel (Section 2.4), they are used here only to map the spatial pattern of change.

Within the Giant Panda National Park, 79.0% of the habitat remained stable in intactness, with 0.5% showing increasing and 0.3% showing decreasing trends; in the Liangshan Mountains, 66.9% remained stable, with 0.5% increasing and 0.5% decreasing. At all five time points (1980, 1990, 2000, 2010, and 2020), the habitat intactness of the Giant Panda National Park was higher than that of the Liangshan Mountains.

### 3.3. Contribution of Disturbance Components to the Spatial Variation in Habitat Intactness

The factor detector showed that the contribution of each disturbance component layer to the spatial variation in habitat intactness followed the order: land-use type (*q* = 0.582, *p* < 0.001) > road construction (*q* = 0.368, *p* < 0.001) > hydropower construction (*q* = 0.124, *p* < 0.001) > water conservancy construction (*q* = 0.059, *p* < 0.001) > mining (*q* = 0.018, *p* = 0.359, not significant; Table 3). Land-use type and road construction were the dominant components of the intactness index and contributed most of its spatial variation.

The interaction detector showed that the joint stratification of any two component layers explained more of the spatial variation in intactness than either layer alone; the combination of land-use type and road construction yielded the highest *q* value (0.768) among all pairwise combinations, indicating that the overlap of these two disturbance components produces the strongest spatial structuring of intactness.

### 3.4. Responses of Giant Pandas to Habitat Intactness

The Mann–Whitney U tests showed that, in all three surveys, the habitat intactness of giant panda occurrence sites in the Liangshan Mountains was significantly higher than that of sites within the national park (second survey, *p* = 0.024; third survey, *p* < 0.001; fourth survey, *p* < 0.001). Given the lower overall background intactness of the Liangshan Mountains, this result suggests that giant pandas concentrate in the remaining high-intactness habitat within this more heavily disturbed landscape.

**Figure 5:**
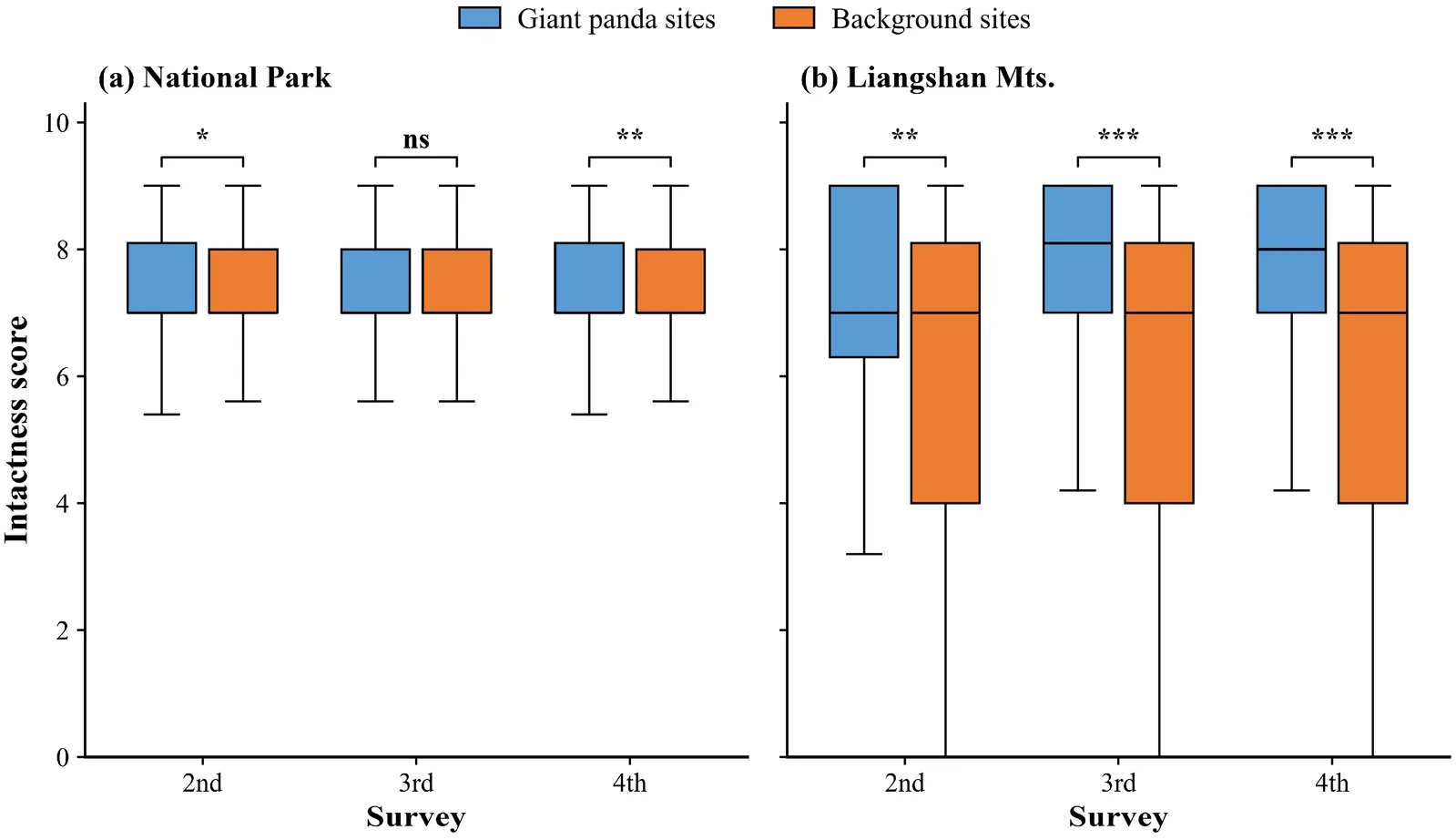
Ecological intactness scores at giant panda occurrence sites versus systematic background points during the 2nd, 3rd, and 4th national giant panda surveys in (**a**) the Giant Panda National Park and (**b**) the Liangshan Mountains. Boxes show the median and interquartile range; whiskers extend to the most extreme values within 1.5× the IQR. * *p* < 0.05, ** *p* < 0.01, *** *p* < 0.001; ns, not significant (Mann–Whitney U test).

Comparisons between giant panda occurrence sites and systematic background points (Figure 5) showed that, within the national park, the intactness of giant panda occurrence sites was significantly higher than that of background points in the second and fourth surveys (*p* = 0.009 and *p* = 0.011, respectively), whereas the difference was not significant in the third survey (*p* = 0.157). In the Liangshan Mountains, occurrence-site intactness was significantly higher than that of background points in all three surveys (all *p* < 0.001). These results indicate that giant panda occurrence is spatially associated with high-intactness habitat.

**Figure 6:**
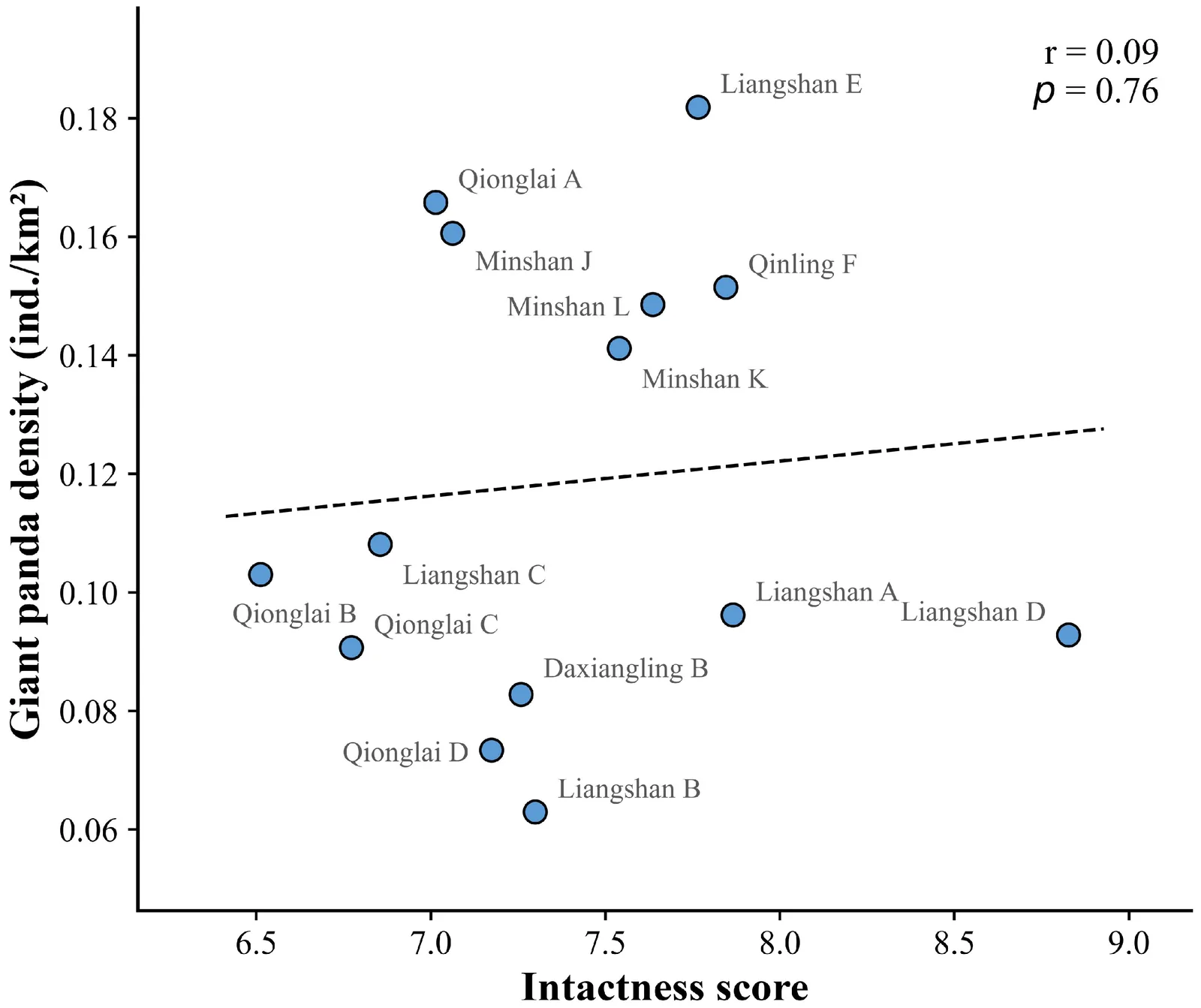
Relationship between habitat ecological intactness score and giant panda density (individuals/km^2^) across local populations. The dashed line is the least-squares linear fit; *r* and *p* are from Pearson correlation analysis.

Pearson correlation analyses showed no significant correlation between habitat intactness and giant panda population density at either the local-population scale (*r* = 0.09, *p* = 0.76, *n* = 14; Figure 6) or the protected-area scale (*r* = −0.26, *p* = 0.15, *n* = 33).

## 4. Discussion

### 4.1. Characteristics of the Spatiotemporal Pattern of Habitat Ecological Intactness

The overall intactness of giant panda habitat in the study area was high; however, 22.4% of the region scored ≤ 6.0 and experienced relatively strong anthropogenic modification. The elevational gradient pattern of intactness is consistent with national-scale findings on land-use change disturbance [24]: low-elevation areas contain settlements, farmland, and transportation corridors and experience stronger cumulative disturbance, whereas high-elevation areas are less accessible and retain greater natural intactness.

Over the 40-year study period, regional intactness declined slowly overall, but most of the habitat remained stable. Intactness increased in some areas, mainly in association with ecological restoration programs such as the Natural Forest Protection Program and the Grain for Green Program [13,42], which reduced deforestation and the encroachment of farmland into forests. The intactness of the Giant Panda National Park was consistently higher than that of the Liangshan Mountains. This difference may be related to the higher human population density, lower household incomes, strong livelihood dependence on forest resources, and long-standing cumulative anthropogenic disturbance pressure in the Liangshan Mountains. Our model did not incorporate diffuse disturbances such as grazing, bamboo shoot collection, and medicinal herb collection, which may have caused intactness in high-elevation areas of the Liangshan Mountains to be overestimated [43,44].

### 4.2. Components Contributing to the Spatial Variation in Habitat Intactness

The Geodetector analysis showed that, among the component layers of the intactness index, land-use change and road construction contributed most to the spatial heterogeneity of habitat intactness. Land-use conversion directly alters the baseline of ecosystems and is jointly regulated by socioeconomic development and national ecological restoration policies. Roads produce well-documented negative ecological effects in giant panda habitat, including fragmenting habitat patches, blocking wildlife movement corridors, reducing bamboo availability along roadsides, and expanding the accessible range of human activities [30,43]; at the global scale, roads are a leading driver of habitat modification and fragmentation in forest ecosystems, and roadless areas are now recognized as a global conservation priority [45,46]. Hydropower and water conservancy projects also reduced local habitat intactness, whereas mining covered a limited spatial extent and showed no significant effect on intactness. Because these component layers enter the intactness index directly (Section 2.5), their *q* values describe relative contributions to the index’s spatial pattern rather than independent causal estimates.

The interaction detector showed that the joint contribution of any two disturbance components exceeded that of either component alone, which is consistent with the fact that anthropogenic disturbances do not act independently: infrastructure construction is often accompanied by land-use change, and their combined footprints drive habitat degradation. Therefore, conservation practice should not target a single disturbance source but should implement integrated management of multiple coexisting anthropogenic stressors.

### 4.3. Responses of Giant Panda Populations to Habitat Intactness Gradients

The intactness of giant panda occurrence sites was generally higher than that of systematic background points. Use–availability comparisons can support a habitat-selection interpretation only when the background sample represents the range of conditions available to the species [47,48]. Our background points instead represent the regional reference condition, and intactness covaries with elevation, land cover, and accessibility; the observed contrast is therefore best interpreted as a spatial association between pandas and low-disturbance habitat, albeit one that is at least partly consistent with selection for intact habitat. This association was more pronounced in the Liangshan Mountains, where disturbance is heavier and giant pandas concentrate in the remaining high-intactness habitat patches [30,49].

However, at the local-population and protected-area scales, there was no significant linear correlation between habitat intactness and population density. This phenomenon can be explained in three ways. First, high-intactness habitat does not necessarily satisfy all the survival requirements of giant pandas, which also depend on slope, bamboo availability, and forest stand structure; some remote high-intactness patches have insufficient bamboo resources and cannot support high population densities [31]. Second, the existing protected-area system in southwestern China has management gaps, boundary overlaps, and differences in enforcement, resulting in inconsistencies between theoretical habitat quality and actual population abundance [26,50,51]. Third, historical legacy disturbances, source–sink effects of metapopulations, and time-lag effects in population recovery [19] further weaken the simple correlation between static intactness indicators and population density.

These results do not negate the conservation value of ecological intactness. Intactness is an important baseline condition of habitat, whereas population density is jointly determined by food resources, topography, metapopulation dynamics, and the effectiveness of protected-area management. Therefore, habitat intactness can serve as one of the core indicators for giant panda habitat assessment but should not be used as the sole predictor of population density.

### 4.4. Implications for Conservation Management

Based on the results of this study, we propose the following management recommendations. First, infrastructure planning in the giant panda distribution range should take into account the disturbance distance-decay relationships quantified here, and new road, hydropower, and mining projects should be kept out of the core giant panda habitat wherever possible. Second, targeted ecological restoration should be carried out for patches with low intactness within key giant panda distribution areas, and built facilities that are incompatible with conservation objectives should be gradually removed to reduce the intensity of cumulative anthropogenic modification. Third, frontline law enforcement in protected areas should be strengthened to mitigate diffuse disturbances such as grazing and excessive bamboo shoot collection [43,44], which are difficult to quantify in intactness modeling. These recommendations are directed primarily at the administrations of the Giant Panda National Park and at the provincial and local forestry and grassland authorities in charge of protected-area management and infrastructure project approval. The first and third measures are mainly institutional and regulatory, so their implementation costs are moderate and largely covered by existing management budgets; the second requires sustained investment in ecological restoration and facility removal, the scale of which can be contained by prioritizing small, well-defined low-intactness patches within key giant panda distribution areas and by implementing restoration in phases.

The Liangshan Mountains harbor multiple vulnerable, isolated small populations, and their habitat intactness is lower than that of the Giant Panda National Park. If the Liangshan Mountains are incorporated into the Giant Panda National Park system in the future [27,29], supporting socioeconomic policies should be prioritized and the coordinated management of multiple anthropogenic disturbance sources implemented, so that regional habitat intactness can gradually rise to a level comparable to that of the existing national park. Incorporating intactness dynamics into systematic conservation planning would help prioritize such boundary expansions [52]. The associated costs are concentrated in livelihood substitution for local communities and in the control of grazing and resource collection, but these costs are likely to be lower than the long-term cost of losing the isolated small populations that such an expansion would protect.

## 5. Conclusions

This study quantitatively assessed the spatiotemporal dynamics of giant panda habitat intactness in the Sichuan section of the Giant Panda National Park and the Liangshan Mountains during 1980–2020. The mean habitat intactness of the study area was 6.70, and 72.0% of the region scored ≥ 7.0; the overall level was high, but a slow declining trend was observed over the past 40 years. Among the disturbance components of the intactness index, land-use type and road construction contributed most to the spatial heterogeneity of intactness. Giant panda occurrence sites were associated with higher-intactness habitat, and this association was particularly pronounced in the more heavily disturbed Liangshan Mountains; however, habitat intactness alone did not predict giant panda population density, because population abundance is jointly regulated by food resources, topography, metapopulation dynamics, and the effectiveness of protected-area management.

Several limitations of this study should be noted. First, the intactness index relies on remotely detectable disturbances, and diffuse activities such as grazing, bamboo shoot collection, and medicinal herb collection—especially prevalent in the Liangshan Mountains and at high elevations—were not quantified, so intactness in these areas may be overestimated. Second, the five intactness time steps (1980–2020) provide only a coarse temporal resolution of change, and the significance classes derived from such short series summarize the spatial pattern of change rather than establish statistical significance in a strict sense. Third, the three national panda surveys differed in spatial precision and were matched to the nearest intactness time layer (Section 2.6), which introduces uncertainty into the population-response comparisons. Fourth, because the explanatory layers entered into Geodetector are components of the intactness index itself, the *q* values describe the relative contribution of these components to spatial variation in intactness rather than independent driving mechanisms. Finally, the background points were systematic grid centers rather than an availability sample designed for habitat-selection inference; accordingly, the panda–intactness relationship is best interpreted as a spatial association rather than demonstrated habitat selection.

Future research should incorporate the diffuse disturbances that were excluded here (Section 2.3) into the intactness assessment system to reduce assessment bias. We also recommend incorporating ecological intactness indicators into the routine monitoring framework of the Giant Panda National Park and its surrounding mountains [25] to provide evidence-based support for the conservation of vulnerable small populations.

## Figures and Tables

**Table 1:** Overview of the main input datasets used in this study.

Dataset	Type	Resolution	Period	Source and Notes
Giant panda occurrence sites	Vector points	—	2nd: 1985–1988; 3rd: 1999–2003; 4th: 2011–2014	National giant panda survey databases and reports (2nd–4th surveys)
Land use and cover (CNLUCC)	Raster	30 m	1980/1990/2000/2010/2020	Resource and Environmental Science Data Center, CAS
Human disturbances (roads, hydropower, water conservancy, mining)	Vector	—	1980–2020	Local management records, survey disturbance registers, and field verification
Digital elevation model (DEM)	Raster	~90 m	—	Used for the elevation-gradient analysis of intactness
Boundaries of local populations and protected areas	Vector	—	4th national survey	Fourth National Giant Panda Survey; 14 populations and 33 protected areas

Note: 2nd, 3rd, and 4th refer to the Second (1985–1988), Third (1999–2003), and Fourth (2011–2014) National Giant Panda Surveys, respectively. CNLUCC, China National Land Use/Cover Change dataset; DEM, digital elevation model; an em dash (—) indicates not applicable.

**Table 2:** Statistical characteristics of habitat ecological intactness in different zones during 1980–2020.

Habitat Ecological Intactness Score (Mean ± SD)					
Zone	1980	1990	2000	2010	2020
Whole study area	6.73 ± 2.06	6.69 ± 2.00	6.69 ± 2.00	6.70 ± 2.00	6.70 ± 2.00
Giant Panda National Park (Sichuan)	7.12 ± 1.72	7.04 ± 1.62	7.03 ± 1.62	7.04 ± 1.61	7.04 ± 1.61
Liangshan Mountains	6.22 ± 2.34	6.25 ± 2.35	6.24 ± 2.34	6.25 ± 2.34	6.26 ± 2.34
Area Proportion of Intactness Trend Categories, 1980–2020 (%)					
Trend Category			Study Area	National Park	Liangshan Mts.
Significant increase			<0.01	<0.01	<0.01
Marginally significant increase			0.02	0.03	<0.01
Non-significant increase			0.44	0.42	0.46
Stable			73.7	79.0	66.9
Non-significant decrease			0.37	0.28	0.49
Marginally significant decrease			<0.01	<0.01	<0.01
Significant decrease			<0.01	<0.01	<0.01

Note: Values in the upper panel are presented as mean ± SD of the habitat ecological intactness index (0–10). The lower panel shows area proportions (%) of seven trend categories classified by the Theil–Sen slope (β) and the Mann–Kendall Z statistic (two-tailed): significant increase/decrease, |Z| > 1.96 (*p* < 0.05); marginally significant increase/decrease, 1.65 < |Z| ≤ 1.96 (0.05 ≤ *p* < 0.10); non-significant increase/decrease, |Z| ≤ 1.65; stable, β = 0. Mts., Mountains.

**Table 3:** *q*-statistics of the factor detector and interaction detector in Geodetector.

Factor Detector					
Component Layer		*q* Statistic		*p* Value	
Land-use type		0.582		<0.001	
Road construction		0.368		<0.001	
Hydropower construction		0.124		<0.001	
Water conservancy construction		0.059		<0.001	
Mining		0.018		0.359	
Interaction Detector *q*(A∩B) (Diagonal: Single-Factor *q*)					
	Land Use	Roads	Hydropower	Water Conserv.	Mining
Land use	0.582				
Roads	0.768	0.368			
Hydropower	0.742	0.494	0.124		
Water conserv.	0.639	0.406	0.170	0.059	
Mining	0.596	0.387	0.144	0.078	0.018

Note: The *q* statistic (0–1) measures the contribution of each disturbance component layer to the spatial heterogeneity of habitat ecological intactness; larger values indicate a stronger contribution. In the lower panel, diagonal entries are single-layer *q* values and off-diagonal entries are interaction *q* values *q*(A∩B) for the corresponding layer pairs. Water conserv., water conservancy construction.

## Data Availability

The land use and cover data were obtained from the Resource and Environmental Science Data Center, Chinese Academy of Sciences. The giant panda survey site data involve conservation-sensitive information on an endangered species and are available from the corresponding authors upon reasonable request.

## Supplementary Materials

The following supporting information can be downloaded at: https://www.mdpi.com/article/10.3390/biology15201799/s1.
