# Supplementary material for: Spatiotemporal Dynamics of Habitat Ecological Intactness and Responses of Wild Giant Panda Populations in Sichuan, China
Source: Biology (Basel). 2026 Oct 9;15(20):1799. doi: 10.3390/biology15201799 (PMC13701200; doi:10.3390/biology15201799)
Supplement: Supplementary file 1 [file biology-15-01799-s001.zip › biology-4606389-supplementary.pdf]

Supplementary Materials

Spatiotemporal Dynamics of Habitat Ecological Intactness and Responses of Wild Giant Panda Populations in Sichuan, China

Qinlong Dai, Junmu Yue, Yue Wang, Xiaodong Gu and Wei Wei

Table S1. Road classification, impact zones, and anthropogenic disturbance intensity scores.

Road class	Road centerline 0–100 m	100–500 m	500–1000 m	1000–3000 m
Expressway	10	3	2	1
Railway	10	3	1	0
First-class road	10	3	2	1
Second-class road	8	2	1	0
Third-class road	6	2	0	0
Fourth-class road	4	1	0	0

Table S2. Hydropower and water conservancy project classification, impact zones, and anthropogenic disturbance intensity scores.

Type	Dam zone 0–500 m	500–1000 m	1000–1500 m	1500–2500 m	2500–5000 m
Small/medium hydropower project	10	2	1	0	0
Large hydropower project	10	8	4	2	1
Extra-large hydropower project	10	8	6	2	1
Small/medium water conservancy project	10	4	2	1	0
Large water conservancy project	10	8	4	2	1
Extra-large water conservancy project	10	8	6	4	1

Table S3. Mining site classification, impact zones, and anthropogenic disturbance intensity scores.

Mine type	Within mine area	0–200 m	200–500 m	500–800 m	800–1000 m
Small mine (<1 km^2 occupied area)	10	4	2	1	0
Large mine (≥1 km^2 occupied area)	10	8	4	2	0

Table S4. Anthropogenic disturbance intensity scores for the second-level classes of the CNLUCC land-use classification.

Category	Second-level class	Description	Intensity score
Forest land	Woodland		1
	Sparse woodland		2
	Shrubland		3
	Plantation	Commercial forest	4
	Plantation	Tea gardens, orchards, etc.	6
Grassland	Grassland		3
Cropland	Dry land		6
	Paddy field		7
Water bodies	Lake		0
	Beach and shoal		0
	Bottomland		0
	Permanent glacier and snow		0
	River and canal		2
	Reservoir and pond		3
Urban, industrial and residential land	Urban land		10
	Rural settlement		10
	Other construction land	Roads, mines, etc.	10
Unused land	Sandy land		0
	Gobi		0
	Saline-alkali land		0
	Marshland		0
	Bare land		0
	Bare rock and gravel		0
	Converted from other types	Natural disturbances such as landslides and debris flows	10

Note: Categories not otherwise specified follow the Land Resources Classification System of China (cited in the main text).
